# DTiGEMS+: drug–target interaction prediction using graph embedding, graph mining, and similarity-based techniques

**DOI:** 10.1186/s13321-020-00447-2

**Published:** 2020-06-29

**Authors:** Maha A. Thafar, Rawan S. Olayan, Haitham Ashoor, Somayah Albaradei, Vladimir B. Bajic, Xin Gao, Takashi Gojobori, Magbubah Essack

**Affiliations:** 1 Computer, Electrical and Mathematical Sciences and Engineering Division (CEMSE), Computational Bioscience Research Center (CBRC), King Abdullah University of Science and Technology (KAUST), Thuwal, Kingdom of Saudi Arabia; 2 Collage of Computers and Information Technology, Taif University, Taif, Kingdom of Saudi Arabia; 3 The Jackson Laboratory for Genomic Medicine, Farmington, CT USA; 4 Faculty of Computing and Information Technology, King Abdulaziz University, Jeddah, Kingdom of Saudi Arabia; 5 Biological and Environmental Sciences and Engineering Division (BESE), King Abdullah University of Science and Technology (KAUST), Thuwal, Kingdom of Saudi Arabia

**Keywords:** Drug repositioning, Drug–target interaction, Machine learning, Graph embedding, Heterogenous network, Similarity-based, Similarity integration, Bioinformatics, Cheminformatics

## Abstract

In silico prediction of drug–target interactions is a critical phase in the sustainable drug development process, especially when the research focus is to capitalize on the repositioning of existing drugs. However, developing such computational methods is not an easy task, but is much needed, as current methods that predict potential drug–target interactions suffer from high false-positive rates. Here we introduce DTiGEMS+, a computational method that predicts Drug–Target interactions using Graph Embedding, graph Mining, and Similarity-based techniques. DTiGEMS+ combines similarity-based as well as feature-based approaches, and models the identification of novel drug–target interactions as a link prediction problem in a heterogeneous network. DTiGEMS+ constructs the heterogeneous network by augmenting the known drug–target interactions graph with two other complementary graphs namely: drug–drug similarity, target–target similarity. DTiGEMS+ combines different computational techniques to provide the final drug target prediction, these techniques include graph embeddings, graph mining, and machine learning. DTiGEMS+ integrates multiple drug–drug similarities and target–target similarities into the final heterogeneous graph construction after applying a similarity selection procedure as well as a similarity fusion algorithm. Using four benchmark datasets, we show DTiGEMS+ substantially improves prediction performance compared to other state-of-the-art in silico methods developed to predict of drug-target interactions by achieving the highest average AUPR across all datasets (0.92), which reduces the error rate by 33.3% relative to the second-best performing model in the state-of-the-art methods comparison.

## Introduction

The exorbitant costs, low success rates, and time-consuming nature of the traditional experiment-based drug discovery processes have led to the incorporation of low cost in silico methods that can fast track drug discovery and development [1]. In this regard, computational methods that predict drug–target interactions (DTIs) have been pursued to reduce the research focus area towards drugs that may be more viable. One of the initial steps in knowing which drugs to pursue is based on the drugs’ ability to interact with a specific target protein to either enhance or inhibit its function [2]. However, there is a limited number of experimentally identified and validated DTI pairs. Thus, DTI prediction is an essential task in the early stage evaluation of potential novel drugs, and the search for novel uses of existing drugs, i.e., drug repurposing [3]. To date, several approaches have been used to predict DTIs, but they all suffer from limitations and require substantially improved prediction performance.

One of the approaches used to predict DTIs, docking simulations [4, 5], requires the 3-dimensional (3D) structure of the protein target. However, such 3D structural information is not available for all targets, which limits the use of this approach. A second approach used to avoid this limitation when predicting DTIs is ligand-based [6, 7]. This approach predicts DTIs by comparing a candidate ligand with the known ligands of the proteins targeted. This approach suffers from low performance in cases where the targeted proteins have few known ligands. Subsequently, several computational methods have been developed to avoid the limitations of these traditional methods. That is, to a certain extent other computational methods may suffer from the same limitation but can incorporate features (such as different drug similarity, statistical and network features from DTIs heterogeneous graph, etc.) beyond ligand interaction features to improve prediction accuracy, and the methods can be designed for target-based drug discovery. Most of these methods use three types of information which are: drug-related information (e.g., chemical information for drugs), target-related information (e.g., protein sequences), or/and known DTI information. These methods can be grouped under three main categories namely: machine learning (ML)-based methods [8–12], deep learning (DL)-based methods [13–16] (DL is a branch of ML), and network-based methods [17–22]. Several comprehensive review articles summarized, analyzed, and compared the methods belonging to these categories [23–29].

ML-based methods were developed using a feature-based approach wherein feature vectors represent DTIs [26] and a similarity-based approach that uses the “guilt-by-association” principle [30]. Some of the first works that successfully predicted DTIs based on supervised ML had been done by Yamanishi and coauthors using pharmacological, chemical, and genomic data [31–33]. Several methods developed based on these assumptions are summarized in [23], and most of these methods achieved promising results. Network-based methods formulate the prediction of DTIs as link prediction problem in a heterogeneous graph [19–21, 34–38]. For example, DASPfind [19] constructs a DTIs graph using a drug–drug similarity matrix, target–target similarity matrix, and known DTIs. After that, DASPfind ranks the DTIs based on their simple path scores to find the top 1% of DTIs. This method outperforms several network-based methods when the single top-ranked predictions are considered using the benchmark DTI datasets, Yamanishi_08 [33]. Since all of the drug–drug similarity (or target–target similarity), as well as DTIs, can be represented as adjacency matrices, matrix factorization approaches have recently been integrated with ML-based methods or/and network-based methods for prediction of DTIs [29, 39–43]. Graph embedding techniques [44, 45] applied on knowledge-graphs also improves the DTI prediction performance [46, 47] through the learning of low-dimensional feature representation of drugs or targets to be used in ML or DL based method. For example, DTINet [20] used matrix factorization as well as graph embedding approaches, to predict a novel DTIs from a heterogeneous graph. That is, DTINet combines several types of drug- and target protein-associated information, including drug-disease association, drug-side effect associations, drug–drug similarity, drug–drug interactions, protein–protein interaction, protein-disease association, and protein–protein similarities to construct a full heterogeneous graph. DTINet constructs the objective function using matrix factorization and then learns a low-dimensional feature representation that captures the topological properties of each node in this heterogeneous graph. DTINet uses this feature representation to predict the DTIs. This method outperforms other state-of-the-art methods using the HPRD and DrugBank datasets. However, DTINet cannot predict the interaction of new drugs or targets, which is considered a limitation of this method [20].

Also, scaling these network-based methods to graphs with a massive number of nodes is not possible. Thus, recent use of DL techniques that are capable of dealing with graphs with a vast number of nodes, as well as large datasets and a large number of features has emerged for prediction of DTIs. These methods use DL techniques in the feature learning step or the prediction step [13, 14, 48–50]. DL-based methods work better with drug and target information from multiple sources for better performance since the information from a single source does not provide sufficient data for DL. For example, NeoDTI (NEural integration of neighbOr information for DTI prediction) [50], a DL-based method, integrates diverse information from 8 different sources (such as drug chemical structure similarity, drug side effects, and protein sequence similarity), to construct a heterogeneous network. NeoDTI learns feature representation for each drug and target by preserving the topological representations. NeoDTI is a powerful and robust tool compared to other recent DTIs prediction methods [50]. Other type of DL-based methods uses raw representations of input data such as SMILES or fingerprints of drugs and amino acid, or nucleotide sequences for proteins to develop an end-to-end learning model to predict DTIs [16, 51–53]. For example, DeepConv-DTI [51] applies a convolutional neural network (CNN) to the amino-acid sequences of proteins and Morgan/Circular fingerprints that is a descriptor of the substructure of a drug after analyzing the molecule as a graph [54]. The CNN captures the local patterns for proteins that enrich their features. After that, the model concatenates the protein and drug features and feeds them to a deep, fully connected layer for the prediction of DTIs.

Here, to further improve prediction performance for DTIs, we propose a computational method that utilizes topological information as well as multiple drug similarities and target similarities. This method called DTiGEMS+ (Drug–target interaction prediction using Graph Embedding, graph Mining, and Similarity-based techniques) approaches DTI prediction as a link prediction problem in a heterogeneous graph. DTiGEMS+ avoided limitations associated with the previously developed methods by integrating different techniques from graph embedding, graph mining, and fusing multiple similarities that reflect different information sources. DTiGEMS+ outperforms several state-of-the-art-methods using benchmark datasets in terms of AUPR performance metric. Our method proves its efficiency in the performance evaluation metrics and in predicting novel DTIs that are validated using literature and different databases.

## Materials

### Benchmark datasets

There are four gold standard datasets (Yamanishi_08) collected and compiled by [33], which were commonly used as benchmark datasets to evaluate the performance of recently developed DTIs prediction methods. Each of the four datasets, namely Enzyme (E), Ion channel (IC), G-protein-coupled receptor (GPCR), and Nuclear receptor (NR), represents one of the significant families of protein targets. These benchmark datasets are publicly available at http://web.kuicr.kyoto-u.ac.jp/supp/yoshi/drugtarge. Table 1 provides the statistics for all datasets used in this study. The sparsity ratio represents the number of known DTIs divided by the number of unknown DTIs and reflects the imbalanced nature between positive and negative samples (see Table 1).

**Table 1:** Benchmark Yamanishi_08 datasets statistics

Statistics	NR	GPCR	IC	Enzyme
No. of drugs	54	223	210	445
No. of targets	26	95	204	664
Known DTIs	90	635	1476	2926
Unknown DTIs	1314	20,550	41,364	292,554
Sparsity ratio	0.068	0.031	0.036	0.010

### Data preprocessing and similarity calculations

**Fig. 1:**
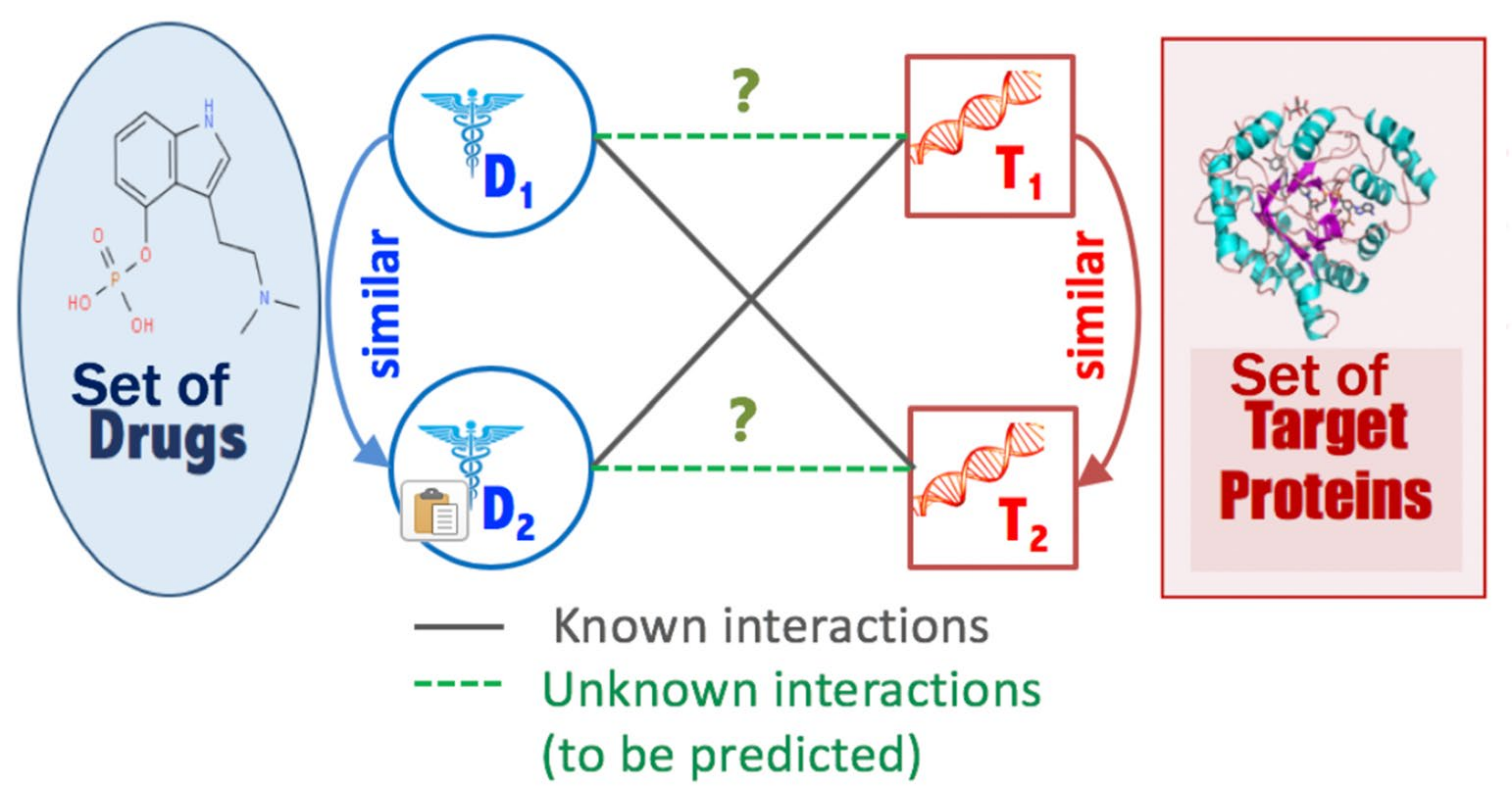
DTIs prediction problem depiction

Starting from the “guilt-by-association” principle that similar drugs may share similar targets and vice versa as illustrated in Fig. 1, we incorporate and utilize several information sources in our approach in the form of different similarity measures (i.e., kernels) between each drug pair or target (i.e., protein) pair. Several drug–drug similarity and target–target similarity are calculated to capture different sources information from different points of view.

#### Multiple drug–drug similarities

Following the [10] study, we computed or retrieved 10 representations or characteristics that can be used to determine drug similarity. That is, six different representations were used for the similarity between drugs based on the chemical structure (SDF, MOL, or SMILES formats) including the SIMCOMP similarity (provided by [33]), and the Spectrum [55], Marginalized [56], Lambda-k [55], Tanimoto, and Min–Max-Tanimoto [57] similarity matrices, calculated using Rchemcpp [58], KEGGREST [59], and ChemmineR [60]. Similarly, three different representations, retrieved from the [10] study, were used for the similarity between drugs based on the side effects, including SIDER [61], AERS-freq [62], and AERS-bit [62] similarity matrices. The tenth drug similarity was calculated based on the gaussian interaction profile (GIP), introduced in [63], that projects the drug–target network structure in the form of a network interaction profile. Additional file 1: Table S1 summarizes all the drug similarity matrices with their names and sources.

#### Multiple target–target similarities

Similar to drug similarities, we computed or retrieved 10 target similarity matrices from the [10] study. Seven different representations mirror the similarity between targets based on the amino-acid protein sequence including the normalized Smith–Waterman (SW) scores [64], and two Spectrum similarity matrices (with k-mers equal to 3 and 4), and four Mismatch similarity matrices (with different parameters of k-mers length and the number of maximal mismatch per k-mer) recalculated using the R packages, KEGGREST [59], and KeBABS [65]. Gene Ontology (GO) similarity matrices based on the GO terms were calculated using the GO.db and annotate R packages [66]. Protein–protein interaction (PPI) similarity that mirrors the shortest distance between each target pair in the PPI network, obtained from [10] study. The GIP is calculated for the targets as we did for the drugs. Additional file 1: Table S1 summarizes all the target similarity matrices with their names and sources.

## Methods

### Problem formulation

In this work, we adopt a network-based approach. We define a weighted heterogeneous graph represented by the DTIs network augmented with the drug–drug similarity graph and target–target similarity graph. This defined graph *G (V, E)* consists of a set of drugs *D* = {*d*_*1*_*, d*_*2*_*,…, d*_*n*_} of n drug nodes, and set of targets *T* = {*t*_*1*_*, t*_*2*_*,…, t*_*m*_} of m target nodes. DTI graph G contains three types of edges. The first type of edge represents the interaction between drug and target nodes, and edges from this type were assigned a weight of 1. The second and third types of an edge represent the similarity between drugs and the similarity between targets, and these types of edges are assigned weights that have a real value between 0 and 1 (0,1]. Given graph G, we define the DTI prediction problem as a link prediction problem, where the goal is to predict unknown true interactions (represented by links) between drugs and targets (see Fig. 1).

We constructed all possible pairs between drugs and targets by generating a “negative sample”. Generating this “negative sample” involved creating connections (i.e., unknown interaction) between drug nodes and target nodes that have no edges. Thus, similar to other existing computational approaches, we used a reliable set of DTIs as positive interactions, and randomly generated drug–target pairs to generate negative DTIs. That is, DTIs existing in the positive set were removed from the randomly generated drug–target pairs to generate negative DTIs. This is done, since there are not enough experimentally-validated negative DTIs available for most sets of drugs and targets. In our work, we believe that random pairing is probably more likely to be well-represented for negative DTIs since the ratios of known (positive) versus non-existing (not known, negative) DTIs is very small. Then, we extracted features for each drug–target pair using different techniques. The feature vector is represented by *X* = {*x*_*1*_*, x*_*2*_*, …, x*_n*m_} and their labels *Y* = {*y*_*1*_*, y*_*2*_*, …, y*_n*m_} where n*m is equal to the number of drugs multiplied by the number of targets that represents the number of all possible (drug, target) pairs. If there is a known interaction for the drug–target pair, the class label y for this pair is equal to 1 (*y *=* 1*); otherwise, the class label is equal to zero (*y *=* 0*). Thus, it is a binary classification task. The aim is to find novel DTIs with high accuracy and low false-positive rate. The proposed method integrates several techniques from the perspective of ML similarity-based, feature-based, and graph-based methods for DTI prediction.

### Similarity-based algorithms

#### Similarity integration technique

**Fig. 2:**
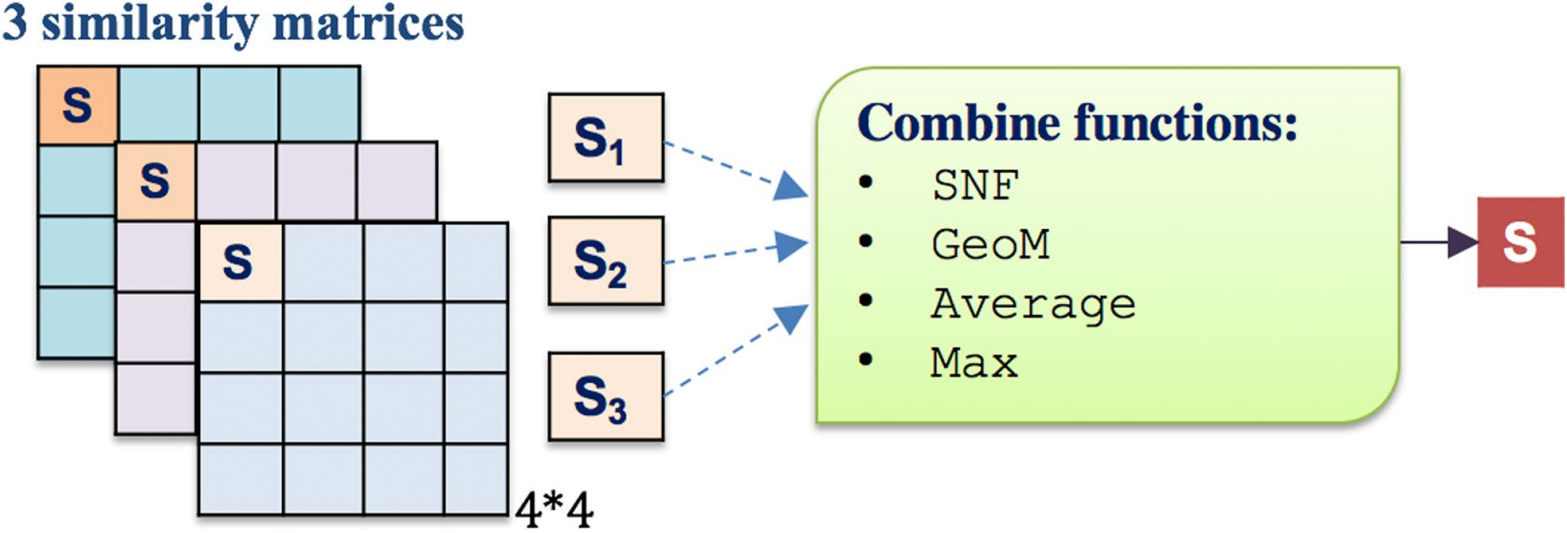
Integrating multiple similarities using different functions

We used several integration functions to combine the multiple similarities matrices, including summing them up to take the average (AVG), taking the geometric mean (GeoM), choosing the maximum similarity value (MAX), or applying the similarity network fusion algorithm (SNF) that was introduced by [67] (see Fig. 2). Each similarity measure is represented by a square matrix, as shown in Fig. 2. The SNF first constructs a sample similarity network for each of the similarity matrices (i.e., drugs represent network nodes, and the similarity represents the networks’ weighted edges but without self-loop edges, and the same thing is done for the target proteins separately). Then, SNF uses a nonlinear method that iteratively integrates these networks by updating each of the networks with the information from the other networks (making the similarity criteria more discriminant with each step) using K-nearest neighbor (KNN). SNF stops when networks converge to a single network after a few iterations. More details about the SNF function and its parameters are explained in [67].

#### Similarity selection technique

To select the optimal subset of similarities that are robust and should improve the prediction task, we applied a forward similarity selection (FSS) procedure as a heuristic process to obtain the best similarity combination. FSS follows the same concept as forward feature selection, where a pair of drug–drug similarity and target–target similarity are added in a “greedy fashion” until one observes no improvement in the performance. In more detail, the input for the FSS algorithm is a list of all drug–drug similarity matrices (all_DDsim) and a list of all target–target similarity matrices (all_TTsim). The algorithm initializes two other lists, one empty list (DDsim) to add selected drug–drug similarity matrix as well as another empty list (TTsim) to add selected target–target similarity matrix. FSS starts with a one drug–drug similarity and one target–target similarity and do this iteratively for all possible combinations of the lists: all_DDsim and all_TTsim and then report the results of all these combinations. The pair of drug–drug similarity and target–target similarity with the best results are chosen to be the first similarity fixed in the DDsim and TTsim. In the second round, we have one fixed drug–drug similarity and target–target similarity, and we add another single similarity to both drug–drug and target–target lists and fuse them using SNF, and report all results. Again, the similarity with the best results is added and fixed in DDsim and TTsim. We repeat these steps, and each round, we add similarities with the best result to the selected similarity sets and fuse them and only stop the repetitions when the results converge (i.e., have no improvements). These “fused” results are used to generate graph ***G1***.

### Graph embedding for feature learning

Given a graph *G* = {*V, E*}, a graph embedding method will transform graph ***G*** into *R*^*d*^ where *d ≪ |v|.* In simple words, the graph embedding method will represent each node in the graph with a feature vector which is much smaller than the actual number of nodes in the graph while preserving the graph structure and properties [45]. To do this, we used the algorithmic framework of node2vec [68], to apply feature representational learning on the full heterogeneous graph G that consists of the training part of known DTIs after hiding the DT edges in the test data, drug–drug similarity matrix (DD sim), and target–target similarity (TT sim) (Fig. 4).

To reduce the node2vec processing time, we removed the weak edges that do not provide any informative meaning, from the drug–drug and target–target similarity graphs. That is, for each drug (or target), we kept the top-k similar drugs (or targets) and removed all other edges. After removing all weak edges, the drug–drug and target–target KNN similarity graphs are augmented with the training part of DTIs and fed into the node2vec model.

After applying node2vec on the heterogeneous graph G to learn feature representation for each drug and target, cosine similarity is calculated between each pair of drugs and each pair of targets to construct two new matrices. These matrices are, M_d_, drug–drug similarity matrix of size n*n where n is the number of drugs, and M_t_, target–target similarity matrix with size m*m, where m is the number of targets; they are used to construct graph ***G2***. After calculating cosine similarity, new edges could appear between pairs of drugs (or targets) based on the structural and topological similarities that don’t have high similarity in the main graph with KNN drug similarity and KNN target similarity, which further prevents the missing of important information.

To utilize and obtain the optimal set of node2vec hyperparameters, grid search algorithm is applied on the validation data. The values of the hyperparameters that are tested on the training data are as follows: Return parameter *p* (controls the likelihood of immediately revisiting a node in the walk) and In–out parameter *q* (allows the search to differentiate between “inward” and “outward” nodes) can be one of the values {0.25, 0.5, 1, 2, 4} as specified in node2vec work, dimension *d* can be {16, 32, 64, 128}, number of walk per source, num-walk tried values {5, 10, 15, 20}, and walk-length takes range based on the size of the graph. For example, in NR dataset we tested values of walk-length starts with 10 and add 5 each time until reach 60 {10:5:60}, while in Enzyme dataset which its graph much bigger we tested the values {50:10:160}. The walk parallelizes by assigning the hyperparameter workers to several workers based on the CPU core number. Additional file 1: Table S4 provides the optimized hyperparameter values for each dataset.

### Graph-based feature extractions for drug–target path scores

At this stage, the two heterogeneous weighted graphs ***G1*** and ***G2*** are used to extract graph-based features. Multiple path scores between each drug–target pair for each graph is used to mirror these features (see Fig. 4). The path score is calculated for each simple path starting from the source node (i.e. drug) and ending with the target node (i.e. target protein) for each drug–target pair using path score, similar to the DASPfind path score introduced by [19] using the following formula:

1 $$score\left( {d_{i} ,t_{j} } \right) = \mathop \sum \limits_{p = 1}^{n} \prod (P_{weights} )$$

where *P *={*p*_*1*_*, p*_*2*_*, …., p*_*n*_} is the set of paths that connect drug_i_ to target_j_. In our study, we reduce the computational costs by limiting the path length to be less than or equal to three (i.e., path length = 2 or 3). Thus, there are six potential path structures Ch = {*C1, C2, C3, C4, C5, C6*} (referred to as path categories in [21, 34]); each starting with a drug node, ending with a target node, and each node in the path appearing only once (no cycling). The six path structures include the two path structures with path length = 2 (C1: (D–D–T) and C2: (D–T–T)), and four path structures with length = 3 (C3: (D–D–D–T), C4: (D–T–T–T), C5: (D–D–T–T), and C6: (D–T–D–T)). We calculated two features for each path structure by determining, 1/the Sum of all meta-path scores for each path structure, and 2/the Max score of all meta-path scores under each path structure. A meta-path is all paths that have the same path structure, and the meta-path score is the product of all the edge weights from the start drug node to the ending target node in the path structure. R_ijh_ denotes the set of paths between a pair of drug_i_ and target_j_. The equations used to determine the features for each path structure are defined and described in Table 2.

**Table 2:** The equations used to determine path structure features

Score description	Equation
The meta-path score is the product of all the edge weight scores from the start drug node to the ending target node in each path structure	$score\left( {d_{i} ,t_{j} ,h, q} \right) = \mathop \prod \limits_{{\forall e_{x} \in P_{q} }} \left( {w_{x} } \right)$
The sum of all meta-path scores for each path structure (Sum feature)	$SumScore\left( {d_{i} ,t_{j} ,h} \right) = \mathop \sum \limits_{{\forall P_{q} \in R_{ijh} }} score\left( {d_{i} ,t_{j} ,h,q} \right)$
The max path score is the highest meta-path score under each path structure (Max feature)	$MaxScore\left( {d_{i} ,t_{j} ,h} \right) = MAX_{{\forall P_{q} \in R_{ijh} }} \left( {score\left( {d_{i} ,t_{j} ,h,q} \right)} \right)$

To ensure longer paths are not disadvantaged in our method, each (Max or Sum) path score is calculated independently, where each score considers all sets of paths that belong to a specific path structure. Thus, scores from different path structures are not mixed together in one feature. Also, scores are further normalized using min max normalization to make sure that features are equally treated by the classifier.

**Fig. 3:**
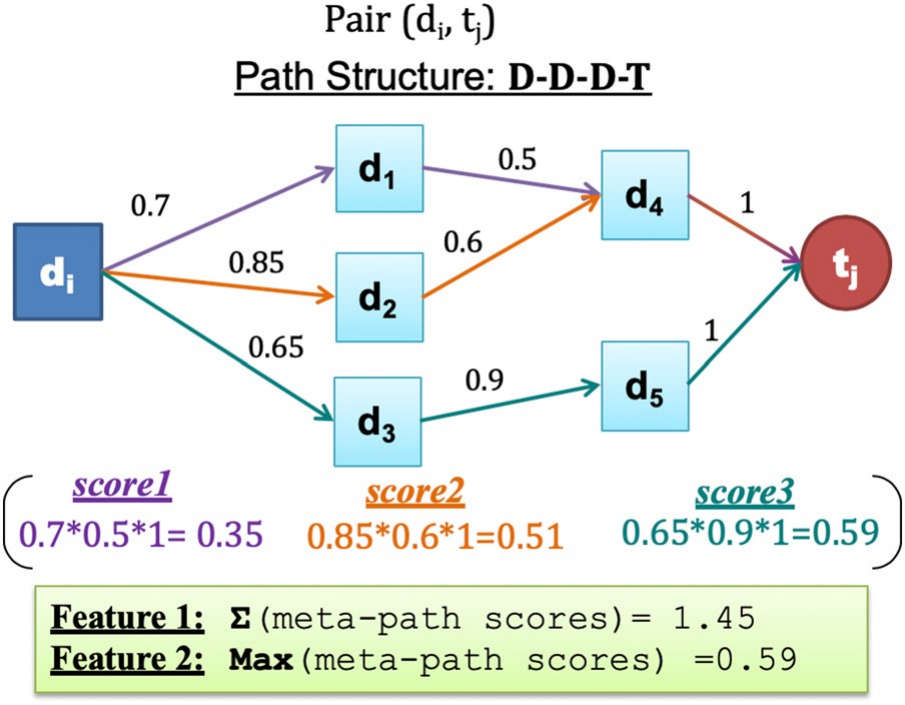
An illustration of Sum and Max scores for a D–D–D–T path structure

We extract 12 features for each (drug, target) pair and for each constructed heterogeneous graph (i.e., ***G1*** and ***G2***) (explained in detail in “DTIs predictive model” section) that are combined to form a 24-dimensional feature vector. Figure 3 provides an example that illustrates the graph-based feature extraction process through the D–D–D–T path structure.

To speed up the running time, we obtain the path scores by applying 3D matrix multiplication. We represented each graph with an adjacency matrix, that includes the drug–drug adjacency matrix (DD_sim), target–target adjacency matrix (TT_sim), and drug–target interaction matrix (DTI). The path score for each path structure is represented by matrix multiplication operation as introduced in [69]. The length of each path structure is equal to the number of multiplied adjacency matrices. Thus, if the path length = 3, such as D–T–T–T, 3 matrices are multiplied to obtain the same results. For Sum score features, regular matrix multiplication is enough to be performed, and the resulting matrix represents the sum features. However, for the Max scores feature, a 3D matrix multiplication is performed to obtain the multiplied value (i.e. the multiplied edge scores) for each path structure, and then choose the max score instead of summation process. Additional file 1: Table S3 provides the corresponding matrix multiplication to each path structure, as well as the semantic meaning for each path structure.

### DTIs predictive model

#### Feature selection

The accuracy of a predictive model relies on identifying the essential features of the examined dataset. Thus, empirical analysis and many experiments were performed (using a concept similar to the forward feature selection method), to identify a collection of the most relevant features for this classification task. Analyzing the performance involved removing one or a combination of features. Consequently, after applying the feature selection step, the dimension of the feature vectors fed into the predictive model reduced from 24 to range between 18 and 20 features based on the dataset.

#### Sampling techniques for imbalanced data

To deal with the number of unknown DTIs being much larger than the number of known DTIs, as shown in Table 1, we applied oversampling techniques on the training data to adjust the data to be balanced. That is, Random oversampling [70] or the Synthetic Minority Over-sampling Technique (SMOTE) [71] were applied to the minority class (i.e., positive known DTIs) to have the same number as the major class (negative unknown DTIs) in training data. The implementation of both techniques was done using the imbalanced-learn python package [72]. Random oversampling contributes to the best classification performance in some datasets, while SMOTE contributes better in other datasets.

#### Classification model

Supervised machine learning model is used to predict DTIs based on three different classifiers for each dataset mainly: Artificial neural network (NN) also called multilayer perceptron (MLP) [73], random forest (RF) [74], and adaptive boosting (Adaboost) [75] classifiers using scikit-learn implementation [76]. In our work, for each classifier used for a specific dataset, the most critical parameters are optimized using the training datasets to improve the classifier performance. Example of these parameters, for the NN classifier, include activation function, the size of hidden nodes and layers, and batch size, while the RF classifier parameters include, the number of trees, the maximum depth of the trees, the number of features to consider when looking for the best split, the function to measure the quality of a split, and others. On the other hand, we used Adaboost to boost the decision tree classifier, so that similar parameters similar to those used in the RF is optimized. The input to these classifiers is the feature vector *X* of all possible drug–target pairs with their labels *Y*.

### The DTiGEMS+ framework

**Fig. 4:**
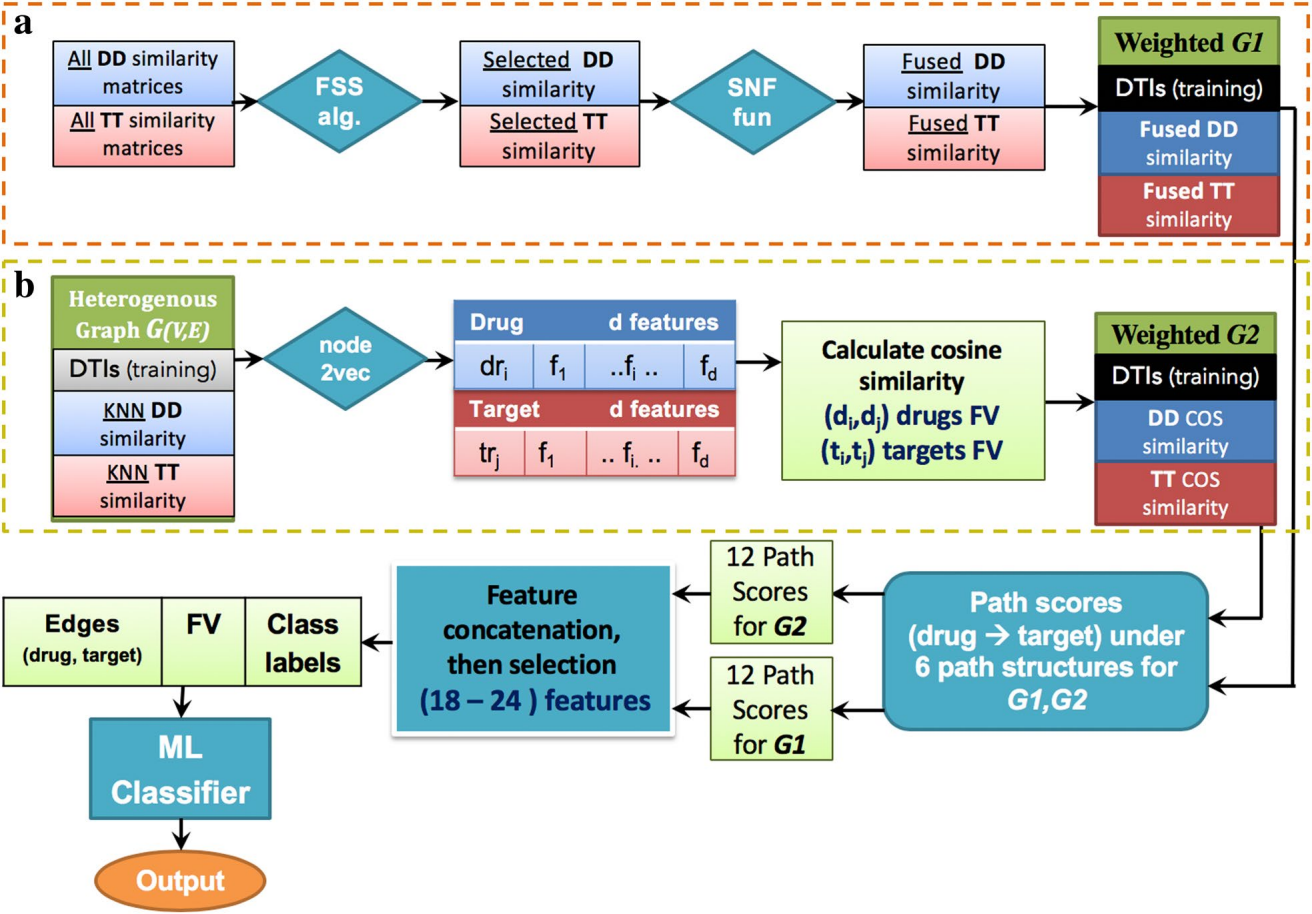
DTiGEMS+ prediction Framework. DTIs: drug–target interactions; DD: drug–drug; TT: target–target; FV: feature vector; FSS alg.: forward similarity selection algorithm; SNF fuc: similarity network fusion function; COS similarity: cosine similarity; ML: machine learning

Figure 4 provides the stepwise framework used to obtain the feature vector, *X*, for all drug–target pairs that are used to predict the missing edges (unknown DTIs to be positive interaction). We generated *X* from two graphs (***G1*** and ***G2***). We generated graph ***G1*** as follows: (**1a**) applied the FSS procedure to all DD and TT similarities, to select the optimal similarities subset, (**2a**) integrated these selected similarities using the SNF algorithm, then, (**3a**) used the DD fused similarity, TT fused similarity, and the DTI training part to construct the heterogeneous graph ***G1***. Simultaneously, we prepared the second graph ***G2*** as follows: (**1b**) applied node2vec to the initial heterogeneous graph G, to generate the feature representations for each node, (**2b**) calculated cosine similarity for each drug–drug pair and target–target pair, then, (**3b**) used the DD cosine similarity, TT cosine similarity, and the DTI training part to construct the heterogeneous graph ***G2***. As a fourth step (**4**), for both graphs ***G1*** and ***G2***, we extracted 12 path scores for each graph, from six path structures. Then as a (**5**) and (**6)** step, feature selection was applied to eliminate weak features, followed by the generated feature vector, *X *= {*x*_*1*_*, x*_*2*_*, …, x*_n*m_}, with their labels *Y* = {*y*_*1*_*, y*_*2*_*, …, y*_n*m_} for all drug–target pairs, being fed into the supervised ML prediction model using NN, RF, or Adaboost classifiers. Then the output of the classifier is the class label, which is either a positive or negative label.

### Evaluation methods

#### Evaluation metrics

To evaluate the prediction accuracy, the area under the receiver operating characteristic (ROC) curve (AUC) [77], as well as the area under the precision-recall curve (AUPR) [77], are calculated on the testing data. To determine the AUC and AUPR, we calculated the false positive rate (FPR), recall (also called true positive rate (TPR) or sensitivity), and precision (also called positive predictive value) [78], based on true positive (TP), false positive (FP), true negative (TN) and false negative (FN) values, as shown in Eqs. 2, 3, and 4, respectively.

2 $$FPR = \frac{FP}{TN + FP}$$

3 $$Recall = TPR = \frac{TP}{TP + FN}$$

4 $$Precision = \frac{TP}{TP + FP}$$

The ROC curve is constructed using different recall, and FPR values of different thresholds, to calculate the AUC. AUPR is calculated based on different precision and recall values at different cut-offs that used to construct the curve, and then the area under this curve is calculated. The closer the value of AUC and AUPR are to 1, the better the performance is. For highly imbalanced (i.e., number of unknown DTIs is much higher than the known DTIs) data, the AUC is considered an over-optimistic evaluation metric for prediction of DTIs, while AUPR is thought to provide better assessment in such imbalance data cases, because it separates the predicted scores of true interactions from the predicted scores of unknown interaction. Thus, we use AUPR as the significant evaluation metric and for the comparison with state-of-the-art methods, but also calculate the error rate (ER), and the relative error rate reduction for the best performing model compared to the second-best performing model (ΔER), defined in Eqs. 5, and 6, respectively:

5 $$ER = 1 - AUPR$$

6 $$\Delta ER = \frac{{\left( {ER_{2} - ER_{1} } \right)}}{{ER_{2} }}$$

#### Experimental settings

For DTiGEMS+ prediction performance evaluation, we performed tenfold cross-validation (CV) on each benchmark dataset separately. The data was randomly partitioned into 10 subsets in a stratified fashion where each subset must include the same percentage of negative and positive samples (i.e., known and unknown DTIs). We kept aside 1 subset of the data for testing and used the remaining 9 subsets to train the model. This process was repeated 10 times to have each subset of the data to be in the test part and the other 9 subsets to train the model. This CV is called a random CV setting where random drug–target pairs are removed to be in test data. The AUPR and AUC calculated for each fold, then the average AUPR and the average AUC of the tenfolds are reported. Here, we removed the corresponding edges to all known DTIs that are in the test set from all constructed graphs in our framework, including ***G***, ***G1***, and ***G2***.

## Results and discussion

Here, we compare the DTI prediction performance between our method and the state-of-the-art methods and validate the newly predicted DTIs using several databases. We also highlighted several possible characteristics that could be boosting the prediction performance of the DTiGEMS+ method compared to other methods.

### DTI prediction performance of DTiGEMS+

To evaluate our method, we compare the DTI prediction performance of DTiGEMS+ and seven state-of-the-art methods using the benchmark Yamanishi_08 datasets. The state-of-the-art methods include TriModel [46], DDR [21], DNLMF [43], NRLMF [39], KronRLS-MKL [10], RLS-WNN [79], and BLM-NII [80]. We chose these methods to give a broad perspective of DTiGEMS+ DTI prediction performance compared to network-based (i.e., graph-based) and or matrix factorization-based methods, as they are all ML similarity-based methods that use prior knowledge to integrate multiple similarity measures from different sources.

To provide a fair comparison of DTI prediction performances, we used the same benchmark datasets, tenfold CV random setting, evaluation metrics, and optimal parameters provided by each method. Our method DTiGEMS+ outperforms all other methods by achieving the best performance across all benchmark datasets (highest averageAUPR = 0.92, highest averageAUC = 0.99), which is 4% higher averageAUPR and 1% higher averageAUC than the second-best method (TriModel) (see Table 3). It also has the best average ranking position across all datasets (the lower ranking position, the better is the method). In Table 3, the best results in each row are indicated in italic font with underline, while the second-best results are only in italic font.

**Table 3:** Average scores for the AUPR, AUC, and ranking position for all comparison methods across all benchmark datasets

Methods	BLM-NII	KronRLS	RLS-WNN	NRLMF	DNILMF	DDR	TriModel	DTiGEMS+
Average AUPR	0.68	0.73	0.76	0.80	0.78	0.87	*0.88*	*0.92*
Average AUC	0.92	0.90	0.96	0.95	0.95	0.96	*0.98*	*0.99*
Average of the ranking position across all datasets	8	7	6	4	5	3	*2*	*1*

We rounded-off all results to two decimal places. The italic font with underline indicates the best result in each category, while the italic font only indicates the second-best result

**Fig. 5:**
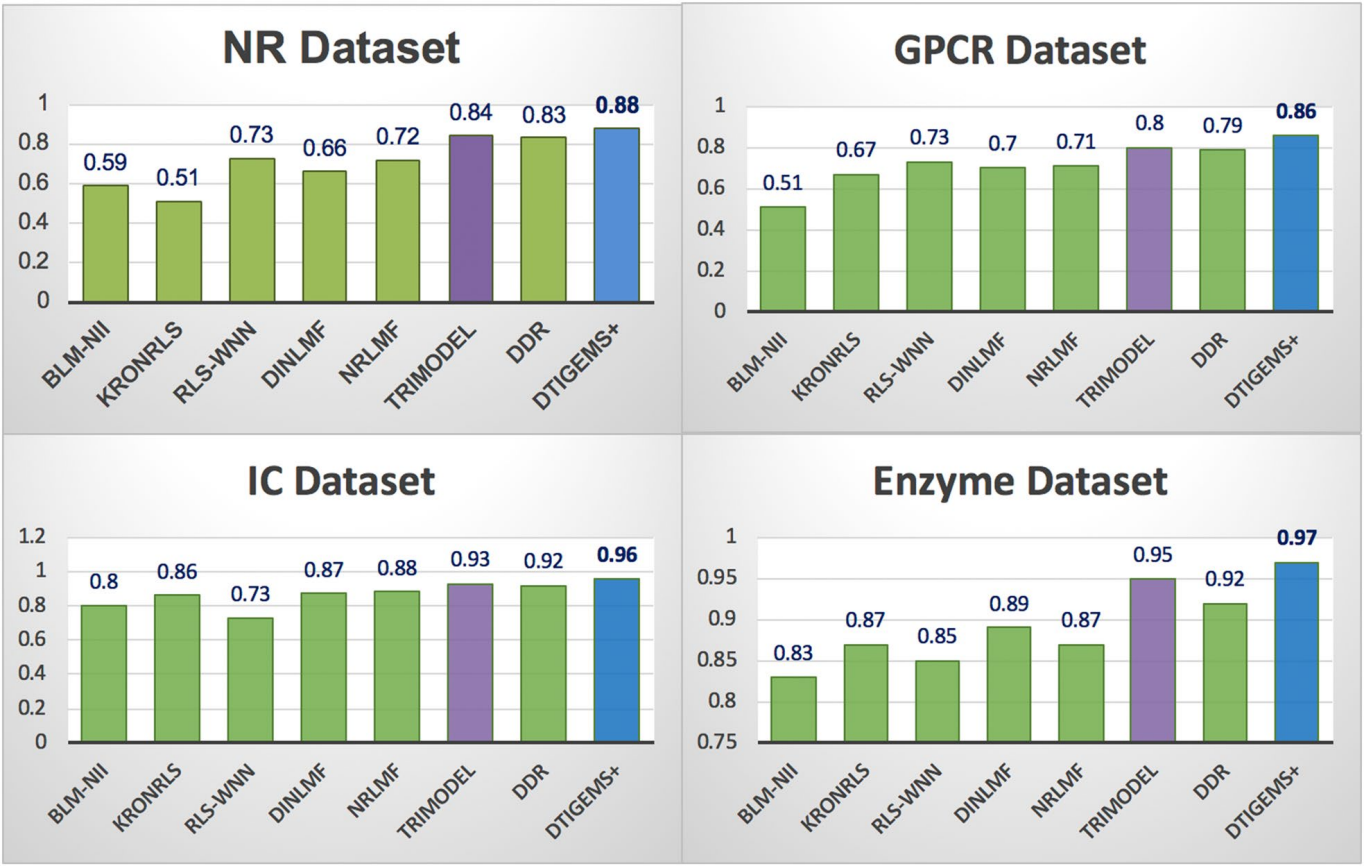
Comparison results for DTiGEMS+ and other methods in terms of AUPR using the Yamanishi_08 datasets. The best performing method is indicated in blue, the second-best method in purple, and all other methods in green

For each dataset, DTiGEMS+ (in blue) performs better in terms of AUPR 0.88(0.094), 0.86(0.031), 0.96(0.013), and 0.97(0.005) for the NR, GPCR, IC, and E datasets, respectively, and the values between brackets are the standard deviations of AUPR in tenfolds CV. DTiGEMS+ outperforming the second-best method (TriModel, in purple) by 4%, 6%, 3%, and 2% for the NR, GPCR, IC, and E datasets, respectively (shown in Fig. 5). DTiGEMS+ also outperformed the other methods in terms of AUC for each dataset except TriModel that have the same performance for the NR, IC, and E datasets (see Additional file 1: Table S5). Figure 5 further shows better DTI prediction performance was achieved using the IC and E datasets; this may be attributed to these datasets having a more extensive set of positive interaction data the models can use to refine the features used for prediction. Moreover, based on individual AUPR values reported from tenfold CV experiments, we calculated the statistical significance in terms of the performance improvement of our method relative to the next best method TriModel using Wilcoxon test which is a nonparametric statistical test that compares two paired groups (refers to the Rank sum test, or the Signed Rank test). As a result, we demonstrate that DTiGEMS+ shows significant statistical difference with probability values (P-values) < 0.05 obtained over GPCR, IC, and E datasets as 0.04, 0.004 and 0.002, respectively, except for NR dataset which has P-value > 0.05.

Two other evaluation metrics are used to gain more insights about the prediction performance improvement of our method DTiGEMS+ over the other methods which are: error rate (ER), and the relative error rate reduction for the best performing method compared to the second-best performing method (ΔER), defined in Eqs. 5 and 6, respectively. Table 4 provides a comparison of the ERs for DTiGEMS+ , as the best performing method, and TriModel, as the second-best performing method. We also provide the relative error rate reduction based on the two top-performing methods in each dataset. The DTiGEMS+ method consistently reduced the relative error rate compared to the other state-of-the-art methods.

**Table 4:** Relative error rates associated with DTiGEMS+ and the second-best performing model TriModel

Datasets	ER_1_ of DTiGEMS+ (%)	ER_2_ of TriModel (%)	Relative ER reduction (%)
*NR*	12.00	16.00	25.00
*GPCR*	14.00	20.00	30.00
*IC*	4.00	7.00	42.86
*E*	3.00	5.00	40.00
The average of ΔER across all datasets			34.47

Furthermore, we show the practical assessment of the predictive power of DTiGEMS+ for real scenarios of DTI prediction at each drug node. This test is done to show the ability of our model in re-positioning a particular drug other than a hub node drug. It should be noted that hub nodes will likely not be the subject of drug research and development as they are likely well-studied. Our procedure goes as follows: first, we calculate the average precision for predicting DTI at each drug, then we average this value over tenfolds. Finally, we calculate mean average precision (MAP) as the mean of tenfolds average precision for each drug across all drug nodes in the graph. We show that DTiGEMS+ archives high MAP values, over NR, GPCR, IC, and E datasets as 0.88, 0.80, 0.91 and 0.88, respectively. Thus, the overall performance of our model is not likely driven by the hub nodes performance.

### DTI prediction and validation of the newly predicted DTIs

To demonstrate the practical use of our model, we assessed its ability to predict the novel DTIs in each of the benchmark datasets separately. The procedure that we follow to predict novel DTIs is as follows: for each dataset, we first trained our model using all known interactions (positive labels) and split the unknown interactions (negative labels) into training and testing sets for each fold in the tenfold CV. In this manner, we determined if any of the unknown DTI (negative labels) are predicted to be positive DTIs, and then ranked the DTIs predicted to be positive, based on their prediction scores. We only reported and validated the novel DTIs that were not part of the training data (i.e., newly predicted DTIs in the testing data).

To verify the novel DTIs, we manually validated the top 10 ranked newly predicted DTIs for each benchmark dataset. We used biomedical literature and several reference databases, including KEGG [81, 82], DrugBank [83, 84], PubChem [85, 86], CheMBLE [87–89], MATADOR [90], SuperTarget [90], Comparative Toxicogenomics Database (CTD) [91, 92], and the annotated database of common toxins and their targets (T3DB) [93]. We found evidence that of the top 10 ranked newly predicted DTIs for each of the 4 benchmark datasets (i.e., for the 40 newly predicted DTIs), 28 DTIs (70%) are known interaction. The interaction data was last updated in 2008; this may be the reason why we managed to verify so many of the newly predicted DTIs. Table 5 shows the top novel DTIs for each dataset with the validation evidence for these validated interactions. However, if there is no evidence found in the literature, we marked the evidence as unknown since there is no proof that this interaction exists.

**Table 5:** Validation of the 10-top ranked newly predicted DTIs for each dataset

Data sets	#	KEGG: Drug ID	Drug name	KEGG: Target ID	Target name	Validation evidence
NR	1	D01132	Tazarotene	hsa6097	RORC (RAR Related Orphan Receptor C)	Unknown
	2	D00182	Norethindrone	hsa2099	ESR1 (Estrogen Receptor Alpha)	PMID: 27245768 T3DB: T3D4745
	3	D00075	Testosterone	hsa5241	PGR (Progesterone Receptor)	PMID: 23229004 PMID: 23933754 C: CHEMBL386630
	4	D01132	Tazarotene	hsa190	NR0B1 (Nuclear Receptor Subfamily 0 Group B Member 1)	Unknown
	5	D00094	Tretinoin	hsa3174	HNF4G (Hepatocyte Nuclear Factor 4 Gamma)	Unknown
	6	D00554	Ethinyl estradiol	hsa2100	ESR2 (Estrogen Receptor 2)	CTD: D004997
	7	D00327	Fluoxymesterone	hsa5241	PGR (Progesterone Receptor)	Unknown
	8	D01294	Ethynodiol diacetate	hsa2100	ESR2 (Estrogen Receptor 2)	Unknown
	9	D00299	Dihydrotachysterol	hsa190	NR0B1 (Nuclear Receptor Subfamily 0 Group B Member 11)	Unknown
	10	D00094	Tretinoin	hsa6095	RORA (RAR Related Orphan Receptor A)	C: CHEMBL38
GPCR	1	D00283	Clozapine	hsa1814	DRD3 (Dopamine Receptor D3)	C: CHEMBL42 M: Clozapine *(direct)* DB: DB00363
	2	D02358	Metoprolol	hsa154	ADRB2 (Adrenoceptor Alpha 1B)	DB: DB00264
	3	D00437	Nifedipine	hsa152	ADRA2C (Adrenergic Receptor alpha-2C)	C: CHEMBL193
	4	D00604	Clonidine hydrochloride	hsa147	ADRA1B (Adrenergic Receptor alpha-1B)	DB: DB00575
	5	D00255	Carvedilol	hsa152	ADRA2C (Adrenergic Receptor alpha-2C)	DB: DB01136
	6	D00451	Sumatriptan	hsa3363	HTR7 (5-Hydroxytryptamine Receptor 7)	Unknown
	7	D00397	Tropicamide	hsa1133	CHRM5 (Cholinergic Receptor Muscarinic 5)	KG: D00397
	8	D00270	Chlorpromazine	hsa152	ADRA2C (Adrenoceptor Alpha 2C)	KG: D00270
	9	D02250	Octreotide acetate	hsa6751	SSTR1 (Somatostatin Receptor 1)	CTD: D015282
	10	D01103	Trospium chloride	hsa1129	CHRM2 (Cholinergic Receptor Muscarinic 2)	KG: D01103
IC	1	D00649	Amiloride hydrochloride	hsa8911	CACNA1I (Calcium Voltage-Gated Channel Subunit Alpha1 I)	M: Amiloride (direct)
	2	D03365	Nicotine	hsa1137	CHRNA4 (Cholinergic Receptor Nicotinic Alpha 4 Subunit)	PMID: 17590520 KG: D03365 DB: DB00184
	3	D00775	Riluzole	hsa2898	GRIK2 (Glutamate Ionotropic Receptor Kainate Type Subunit 2)	KG: D00775
	4	D00438	Nimodipine	hsa779	CACNA1S (Calcium Voltage-Gated Channel Subunit Alpha1S)	KG: D00438 DB: DB00393
	5	D00726	Metoclopramide	hsa1138	CHRNA5 (Cholinergic Receptor Nicotinic Alpha 5 Subunit)	Unknown
	6	D00552	Benzocaine	hsa6331	SCN5A (Sodium Voltage-Gated Channel Alpha Subunit 5)	KG: D00552
	7	D00542	Halothane	hsa3736	KCNA1(Potassium Voltage-Gated Channel Subfamily A Member 1)	Unknown
	8	D02098	Proparacaine hydrochloride	hsa8645	KCNK5 (Potassium Two Pore Domain Channel Subfamily K Member 5)	Unknown
	9	D01599	Gliclazide	hsa3758	KCNJ1 (Potassium Inwardly Rectifying Channel Subfamily J Member 1)	Unknown
	10	D00538	Zonisamide	hsa6331	SCN5A (Sodium Voltage-Gated Channel Alpha Subunit 5)	DB: DB00909 KG: D00538
E	1	D00437	Nifedipine	hsa1559	CYP2C9 (Cytochrome P450 Family 2 Subfamily C Member 9)	CTD: D009543 PMID: 9929518
	2	D00574	Aminoglutethimide	hsa1589	CYP21A2 (Cytochrome P450 Family 21 Subfamily A Member 2)	M: Aminoglutethimide *(indirect)* PMID: 8201961
	3	D00410	Metyrapone	hsa1583	CYP11A1 (Cytochrome P450 Family 11 Subfamily A Member 1)	CTD: D008797
	4	D00437	Nifedipine	hsa1585	CYP11B2 (Cytochrome P450 Family 11 Subfamily B Member 2)	M: Nifedipine- *(indirect)* CTD: D009543
	5	D00410	Metyrapone	hsa1543	CYP1A1 (Cytochrome P450 Family 1 Subfamily A Member)	PMID: 9512490
	6	D03670	Deferoxamine	hsa51302	CYP39A1 (Cytochrome P450 Family 39 Subfamily A Member 1)	Unknown
	7	D00043	Isoflurophate	hsa1991	ELANE (Elastase, Neutrophil Expressed)	M: Diisopropylfluorophosphate (indirect)
	8	D00947	Linezolid	hsa4129	MAOB (Monoamine Oxidase B)	CTD: D000069349
	9	D03670	Deferoxamine	hsa4353	MPO (Myeloperoxidase)	M: Desferrioxamine (indirect)
	10	D05458	Phentermine	hsa4128	MAOA (Monoamine Oxidase A)	KG: D05458 DB: DB00191

C: ChEMBL; CTD: comparative toxicogenomics database; DB: DrugBank; M: MATADOR; KG: KEGG; PMID: PubMed; T3DB: toxin and toxin–target database

### Distinctive characteristics of DTiGEMS+

Table 4 and Fig. 4 show that DTiGEMS+, TriModel, and DDR are the three top-performing methods, respectively, and all three methods are graph-based. Being graph-based allows these methods to avoid some of the limitations associated with the other methods, and they have a few common characteristics that boost their performance. The main characteristics of these methods are that they formulate the problem as a link prediction in a heterogeneous graph, so they constructed the heterogeneous graph through the integration of multiple information types from different sources. DDR constructed the heterogeneous graph through the integration of multiple similarities from different sources of information, while the TriModel used knowledge graph embedding to infer novel DTIs. DTiGEMS+, on the other hand, kind of fused these methods, by constructing one heterogeneous graph (***G1***) through the integration of multiple similarities from different sources of information and a second graph (***G2***) using cosine similarity based on node embeddings generated by applying node2vec on the initial DTI graph (***G***).

Both DTiGEMS+ and DDR integrating multiple similarities should yield a significant improvement in the prediction task. However, some similarities are weak, which means they introduce noise into the data along with the vital information used in the learning and prediction processes. Thus, instead of integrating all similarities, DTiGEMS+ and DDR used similarity selection to identify the optimal subset of similarities that gives optimal results while eliminating the noise. In this regard, DTiGEMS+ used the FSS algorithm (explained in “Similarity-based algorithms” section) to provide useful insights into the optimal subset of similarities for drugs, as well as for target. This algorithm continues to add similarities and only stops when further improvements are no longer visible. Thus, this procedure is time-consuming but provide a higher probability of determining the optimal subset of similarities. On the other hand, to select the optimal similarity subset, DDR calculated entropy values that indicate if the information carried by the similarity matrix is less or more random, then implemented a cut-off to remove similarity matrices carrying weak or random information. The issue here is that even though DDR produced excellent results, the cut-off used could have removed similarity matrices that contain information that contributed to the better performance of DTiGEMS+.

After selecting the optimal subset of similarities, both DTiGEMS+ and DDR used an integration function to integrate the similarities. In “Similarity-based algorithms” section, we showed that SNF is the better performing integration function for all datasets, while the AVG function performed the second-best for most datasets except the GPCR dataset, where its performance is identical to SNF. Both DTiGEMS+ and DDR implemented SNF, which not only integrates the similarities but also enforces noise reduction as part of the integration process. That is, the low-weight edges that represent weak similarity have disappeared, captures the most informative features. Thus, the better performance seen with both DTiGEMS+ and DDR compared to other methods, may also be contributed to by the implementation of SNF, which is the only integration function that enforces noise reduction. Additional file 1: Table S2 provides the set of drug–drug similarities as well as the set of target–target similarities that are selected and then fused, as well as the best-performing integration function/s.

For DTiGEMS+, the KNN that performs noise reduction is not only a component of SNF, we also used KNN (on the drug–drug similarity, target–target similarity) augmented with DTI to construct the graph fed to node2vec. In this manner, the graph used for generating the embeddings needed to construct graph ***G2*** only provides the informative edges for the generation of good quality graph embeddings that capture meaningful proximity information between nodes. Another advantage of applying node2vec on the graph that kept just the KNN similar drugs and targets, is that it reduced the node2vec model running time since the number of edges for each drug similarity graph (and target similarity graph) reduced from *n (n *− *1)/2* to (K*n) where n is the number of drugs. Second, we computed two cosine similarity matrices based on node2vec feature representations for each drug pair and target pair because it gives unique similarity between nodes that carry meaningful topological, relational, and structural information. So, even if the two similar nodes are not close based on the Euclidean distance, their feature vectors could still have a small angle between them, indicating their high similarity. Formulating a new graph with these new similarities provided a better representation of the graph that was used to extract the path score features. These factors may provide DTiGEMS+ with an advantage over the TriModel, as they may be contributing to the capturing of quality embeddings due to noise reduction and or our method identifying potential DTIs excluded from TriModel. It is important to mention that we did the experiments by feeding the whole graph without removing any edge to node2vec and the results of AUPR were close to or lower than the experiment results when we used KNN drugs similarity and KNN targets similarity which means removing weak edges is not causing that we are missing important information.

DTiGEMS+ has another advantage over other graph-based methods that used path structure scores as their model features, such as in [21, 34]. We analyzed these features and recognized that the D–T–D–T path structure, for example, is not based on informative features. That is, the D–T–D–T path structure is generated only using the information of known DTIs, which is limited in number, causing these features to be sparse. So, we removed the sum and max features for such path structure for both graph ***G1*** and ***G2***.

At the classification stage, some other methods directly apply RF as it is a recognized prediction tool that runs efficiently on large datasets, and is less prone to overfitting. However, for DTiGEMS+, we accessed the performance of three different classifiers (RF, NN, Adaboost) on each dataset, then chose the best performing classifier for each dataset. NN classifier performed the best for the NR dataset. We expected this result as the NN classifier is known to perform better when modeling high volatility data, which is the case for the NR dataset due to its small size. Nonetheless, ensemble learning techniques such as RF and Adaboost have proven efficacy when dealing with DTI prediction problems [8, 21, 34, 94, 95]. The RF classifier combines several individual classifiers that vote and nominates the majority voting class as the prediction class. On the other hand, the Adaboost classifier creates a robust classifier from several weak classifiers by building a first model from the training data, and then create a second model that tries to correct the errors in the first model; this process is repeated until the prediction performance of the training data is improved. One advantage of RF over Adaboost is that RF runs in parallel while Adaboost runs sequentially, so RF is a much faster classification process. Nonetheless, Adaboost performance was very close to NN for the NR dataset (less by 1% in AUPR). Moreover, Adaboost performed better than both RF and NN for the other datasets (GPCR, IC, and E). It is worth noting that the RF classifier was, however, competitive for IC and E datasets (very close AUPR) with a more significant number of known interactions.

## Conclusion

Our work introduced a novel computational method for drug–target interactions prediction named (DTiGEMS+). DTiGEMS+ integrated different techniques from ML, graph embedding, graph mining, and similarity-based methods. That is, (1) graph embedding was used in node2vec feature representation to benefit from the network topology and structural features, (2) graph mining was used to extract path score features, (3) similarity-based techniques were used to select and integrate multiple similarities from different information sources, and finally, (4) ML for classification. The novelty of our method lies in generating graph-based path score features from two graphs that were constructed using the same DTIs but using different types of similarity matrices that carry unique information. For example, Graph ***G1*** used to fuse the drug–drug and target–target similarities carry complementary information from chemical structure and side effects for drugs, etc., and gene ontology and amino-acid sequences for target proteins, etc., while graph, ***G2*** used drug–drug and target–target cosine similarities of generated embedding that carry topological information. DTiGEMS+ proved its efficiency by outperforming seven state-of-the-art methods using several evaluation metrics, and by predicting novel DTIs that were validated using published literature and different online databases.

For further improvements to DTiGEMS+ , we suggest applying different embeddings techniques, integrating more similarity measures from more sources, and generating more graph-based features. Also, as the current implementation of DTiGEMS+ constructs negative DTIs from the random pairing of drugs and targets that have no edges (unknown interaction), in the future, we plan to extend the functionality of our method to create a reliable set of negative DTIs following [96]. Furthermore, we intend to use our method to predict DTIs for new drugs or new targets. Some potential extensions of our work include applying DTiGEMS+ to different graphs (i.e., network) formulated as a link prediction problem. Popular examples of link prediction in the bioinformatics field include but are not limited to, drug–drug interactions prediction, drug-disease interactions prediction, gene-disease association prediction. Another extension would be amending DTiGEMS+ to address DTIs as a regression problem for the prediction of the binding affinity between drugs and their target proteins.

## Supplementary information

**Additional file 1.** Additional Tables.

## Data Availability

The source code and datasets used in the paper can be found in the: https://github.com/MahaThafar/Drug–Target–Interaction–Prediciton-Method.

## Abbreviations

DTIs: Drug–target interactions
3D: 3-dimensional
ML: Machine learning
DL: Deep learning
CNN: Convolutional neural network
E: Enzyme
IC: Ion channel
GPCR: G-protein-coupled receptor
NR: Nuclear receptor
GIP: Gaussian interaction profile
SW: Smith–Waterman
GO: Gene ontology
PPI: Protein–protein interaction
SNF: Similarity network fusion
KNN: K-nearest neighbor
FSS: Forward similarity selection
DD sim: Drug–drug similarity matrix
TT sim: Target–target similarity matrix
SMOTE: Synthetic Minority Over-sampling Technique
NN: Neural network
MLP: Multilayer perceptron
Adaboost: Adaptive boosting
RF: Random forest
ROC: Receiver operating characteristic
AUC: The area under ROC curve
AUPR: The area under the precision-recall curve
FPR: False positive rate
TPR: True positive rate
ER: Error rate
CV: Cross validation
MAP: Mean average precision
P-value: Probability value
