# Supplementary material for: DTiGEMS+: drug–target interaction prediction using graph embedding, graph mining, and similarity-based techniques
Source: J Cheminform. 2020 Jun 29;12:44. doi: 10.1186/s13321-020-00447-2 (PMC7325230; doi:10.1186/s13321-020-00447-2)
Supplement: Supplementary file 1 — Additional file 1. Additional Tables. [file 13321_2020_447_MOESM1_ESM.docx]

**DTiGEMS+: Predicting Drug-Target Interactions integrating Graph Embedding, Graph Mining, and Similarity-based Techniques**

**Additional File 1**

Maha A. Thafar ^(1)(2)^, Rawan S. Olyan ^(1)(3)^, Somayah Albaradei ^(1)(4)^, Haitham Ashoor ^(1)(3)^, Vladimir B. Bajic ^(1)^, Xin Ga ^(1)^, Takashi Gojobori ^(1)(5)^, Magbubah Essack ^(1)*^

*To whom correspondence should be addressed.

**Supplementary Tables**

**Supplementary Table S1: Drugs and Targets similarity names, types, and sources**

| **Data Type** | **Similarity type and name** | **Similarity Source** |
| --- | --- | --- |
| **Drugs** | *Aers-bit: adverse event report systems* | Side-effect |
|  | *Aers-freq: adverse event report systems* | Side-effect |
|  | *Lambda: lambda k-kernel* | Chemical structure |
|  | *Marginalized* | Chemical structure |
|  | *MinmaxTanimoto* | Chemical structure |
|  | *Sider* | Side-effect |
|  | *Simcomp (Graph kernel)* | Chemical structure |
|  | *Spectrum* | Chemical structure |
|  | *Tanimoto* | Chemical structure |
|  | *GIP: Gaussian Interaction Profile* | Drug target interactions network |
| **Target** **Proteins** | *GO: Gene Ontology Semantic Similarity* | Function Annotation |
|  | *Mismatch-n-k3m1* | Amino-acid Sequence |
|  | *Mismatch-n-k3m2* | Amino-acid Sequence |
|  | *Mismatch-n-k4m1* | Amino-acid Sequence |
|  | *Mismatch-n-k4m2* | Amino-acid Sequence |
|  | *PPI: proximity in protein-protein interaction* | Protein-protein interactions network |
|  | *Spectrum-n-k3* | Amino-acid Sequence |
|  | *Spectrum-n-k4* | Amino-acid Sequence |
|  | *SW-n: normalized Smith Waterman alignment score* | Amino-acid Sequence |
|  | *GIP: Gaussian Interaction Profile* | Drug target interactions network |

**Supplementary Table S2: Integration functions and optimal similarities subset for each benchmark dataset that after applying the similarity selection procedure.**

| **Datasets** | **Fusion function** | **Selected drug-drug similarities set** | **Selected target-target similarities set** |
| --- | --- | --- | --- |
| NR | SNF | *Simcomp, Ares-freq, Marginalized* | *SW-n, GO, Spectrum-n-k4* |
| GPCR | SNF/ AVG | *Simcomp, Aers-bit, Sider, Aers-freq* | *GIP, SW-n, Spectrum-n-k3, GO* |
| IC | SNF | *GIP, Simcomp, Aers-bit, Sider, Aers-freq* | *GIP, SW-n, Spectrum-n-k4, GO, PPI* |
| Enzyme | SNF | *GIP, Aers_bit, Simcomp, Sider, Aers-freq* | *GIP, GO, SW-n, Spectrum-n-k4, PPI* |

**Supplementary Table S3: Best obtained node2vec hyperparameters using the grid search.**

| **node2vec Parameters** | **NR** | **GPCR** | **IC** | **Enzyme** |
| --- | --- | --- | --- | --- |
| Number of features: dimensions --d | 16 | 32 | 128 | 64 |
| Length of walk per source: --walk-length | 50 | 20 | 50 | 100 |
| Return hyperparameter: -- p | 0.25 | 0.5 | 0.5 | 1 |
| In-out hyperparameter: --q | 0.5 | 2 | 0.25 | 1 |
| Number of walks per source: --num-walk | 10 | 10 | 10 | 10 |

**Supplementary Table S4: path structures with corresponding matrix multiplications, and the semantic meanings.**

| **Path structure** | **Semantic Description** | **Corresponding matrix multiplication** | **Length** |
| --- | --- | --- | --- |
| D-->D-->T | Drug is *similar* to drug *interact* with target | DD_sim * DTI | 2 |
| D-->T-->T | Drug *interact* with target *similar* to target | DTI * TT_sim | 2 |
| D-->D-->D-->T | Drug is *similar* to drug *similar* to drug *interact* with target | (DD_sim * DD_sim) * DTI | 3 |
| D-->T-->T-->T | Drug *interact* with target *similar* to target *similar* to target | DTI * (TT_sim * TT_sim) | 3 |
| D-->D-->T-->T | Drug is *similar* to drug *interact* with target *similar* to target | DD_sim * DTI * TT_sim | 3 |
| D-->T-->D-->T | Drug *interact* with target *interact* with drug *interact* with target | DTI * DTI.Transpose * DTI | 3 |

**Supplementary Table S5:  The AUC scores for all methods for each benchmark dataset separately** (All results rounded to 2 digits. Bold fonts with underline indicate best results while bold fonts indicate second best).

| **Dataset** | **AUC of each method** | | | | | | | |
| --- | --- | --- | --- | --- | --- | --- | --- | --- |
|  | BLM-NII | KronRLS | RLS- WNN | NRLMF | DNILMF | DDR | TriModel | DTiGEMS+ |
| ***NR*** | 0.91 | 0.87 | 0.93 | 0.93 | 0.92 | 0.93 | **0.97** | **0.97** |
| ***GPCR*** | 0.88 | 0.91 | 0.95 | 0.95 | 0.96 | 0.96 | **0.98** | **0.99** |
| ***IC*** | 0.91 | 0.90 | 0.98 | 0.98 | 0.94 | 0.98 | **0.99** | **0.99** |
| ***Enzyme*** | 0.96 | 0.93 | 0.96 | 0.95 | 0.96 | 0.97 | **0.99** | **0.99** |
